# Structure-inclusive similarity based directed GNN: a method that can control information flow to predict drug–target binding affinity

**DOI:** 10.1093/bioinformatics/btae563

**Published:** 2024-09-18

**Authors:** Jipeng Huang, Chang Sun, Minglei Li, Rong Tang, Bin Xie, Shuqin Wang, Jin-Mao Wei

**Affiliations:** Centre for Bioinformatics and Intelligent Medicine, Nankai University, Tianjin 300071, China; College of Computer Science, Nankai University, Tianjin 300071, China; Tianjin Key Laboratory of Network and Data Security, Tianjin 300350, China; Centre for Bioinformatics and Intelligent Medicine, Nankai University, Tianjin 300071, China; College of Computer Science, Nankai University, Tianjin 300071, China; Tianjin Key Laboratory of Network and Data Security, Tianjin 300350, China; Centre for Bioinformatics and Intelligent Medicine, Nankai University, Tianjin 300071, China; College of Computer Science, Nankai University, Tianjin 300071, China; Tianjin Key Laboratory of Network and Data Security, Tianjin 300350, China; Centre for Bioinformatics and Intelligent Medicine, Nankai University, Tianjin 300071, China; College of Computer Science, Nankai University, Tianjin 300071, China; Tianjin Key Laboratory of Network and Data Security, Tianjin 300350, China; College of Computer and Cyber Security, Hebei Normal University, Shijiazhuang 050024, China; College of Computer and Information Engineering, Tianjin Normal University, Tianjin, Xi Qing District 300387, China; Centre for Bioinformatics and Intelligent Medicine, Nankai University, Tianjin 300071, China; College of Computer Science, Nankai University, Tianjin 300071, China

## Abstract

**Motivation:**

Exploring the association between drugs and targets is essential for drug discovery and repurposing. Comparing with the traditional methods that regard the exploration as a binary classification task, predicting the drug–target binding affinity can provide more specific information. Many studies work based on the assumption that similar drugs may interact with the same target. These methods constructed a symmetric graph according to the undirected drug similarity or target similarity. Although these similarities can measure the difference between two molecules, it is unable to analyze the inclusion relationship of their substructure. For example, if drug A contains all the substructures of drug B, then in the message-passing mechanism of the graph neural network, drug A should acquire all the properties of drug B, while drug B should only obtain some of the properties of A.

**Results:**

To this end, we proposed a structure-inclusive similarity (SIS) which measures the similarity of two drugs by considering the inclusion relationship of their substructures. Based on SIS, we constructed a drug graph and a target graph, respectively, and predicted the binding affinities between drugs and targets by a graph convolutional network-based model. Experimental results show that considering the inclusion relationship of the substructure of two molecules can effectively improve the accuracy of the prediction model. The performance of our SIS-based prediction method outperforms several state-of-the-art methods for drug–target binding affinity prediction. The case studies demonstrate that our model is a practical tool to predict the binding affinity between drugs and targets.

**Availability and implementation:**

Source codes and data are available at https://github.com/HuangStomach/SISDTA.

## 1 Introduction

Drug development usually involves steps such as molecular design, preclinical studies, and clinical trials, which are time-consuming and costly. Studies have shown that the average success rate for developing a new molecular entity is only 2.01% and that the clinical development cycle takes an average of 13.9 years (Yeu *et al.* 2015). To reduce the cost of drug development, biologists try to find new indications for approved drugs (i.e. drug repurposing). Nearly 70 existing FDA-approved drugs are currently being investigated to see if they can be repurposed to treat COVID-19 (Gordon *et al.* 2020). The short cycle time and low cost of drug repurposing shorten the review process and have significant implications for drug development. One of the key steps in drug repurposing is the identification of drug−target relationships.

Computational methods are considered for predicting reliable drug−protein interactions to reduce the workload of subsequent experiments for drug−target relationship identification. With the continuous development of biological big data, machine learning methods have been applied to various practical tasks in the field of bioinformatics. Determining the new drug−target relationship with computational methods has become a research hotspot in the field of drug development (Eisenstein 2022).

Drug−target relationship prediction algorithms can be broadly classified into two categories. One class focuses on drug–target interaction (DTI) prediction, which treats the prediction task as a binary classification task (Li *et al.* 2022, Sun *et al.* 2022). For example, AEFS (Sun *et al.* 2021) tried to predict DTIs by maintaining the consistency between drug properties and their functions with a multilayer encoder. Luo *et al.* (2017) and Zeng *et al.* DNNCC (Sun *et al.* 2023) projects the original features of drugs and proteins into the same embedding space to ensure their semantic consistency. There is also a body of work (Cheng *et al.* 2012, Lu *et al.* 2017) that constructs drug−drug or drug−protein networks by exploiting similarities, to learn potential information from close neighbors.

Another class of approaches treats drug−target relationship prediction as a regression task, considering the drug−target binding affinity (DTA) to contain more information (Thafar *et al.* 2019). SimBoost (He *et al.* 2017) used a similar similarity matrix and proposed a novel nonlinear approach to predict DTA with gradient-boosting regression trees. GraphDTA (Nguyen *et al.* 2021) tried to treat compounds as graphs and used multiple graph neural networks (GNN) to extract features from them for prediction. Zeng *et al.* (2021) established a model with self-attention mechanism and multi-headed attention mechanism for relationship perception. MGraphDTA (Yang *et al.* 2022) considered protein tertiary structure and used deep graph convolution to get better predictions.

**Figure 1.**
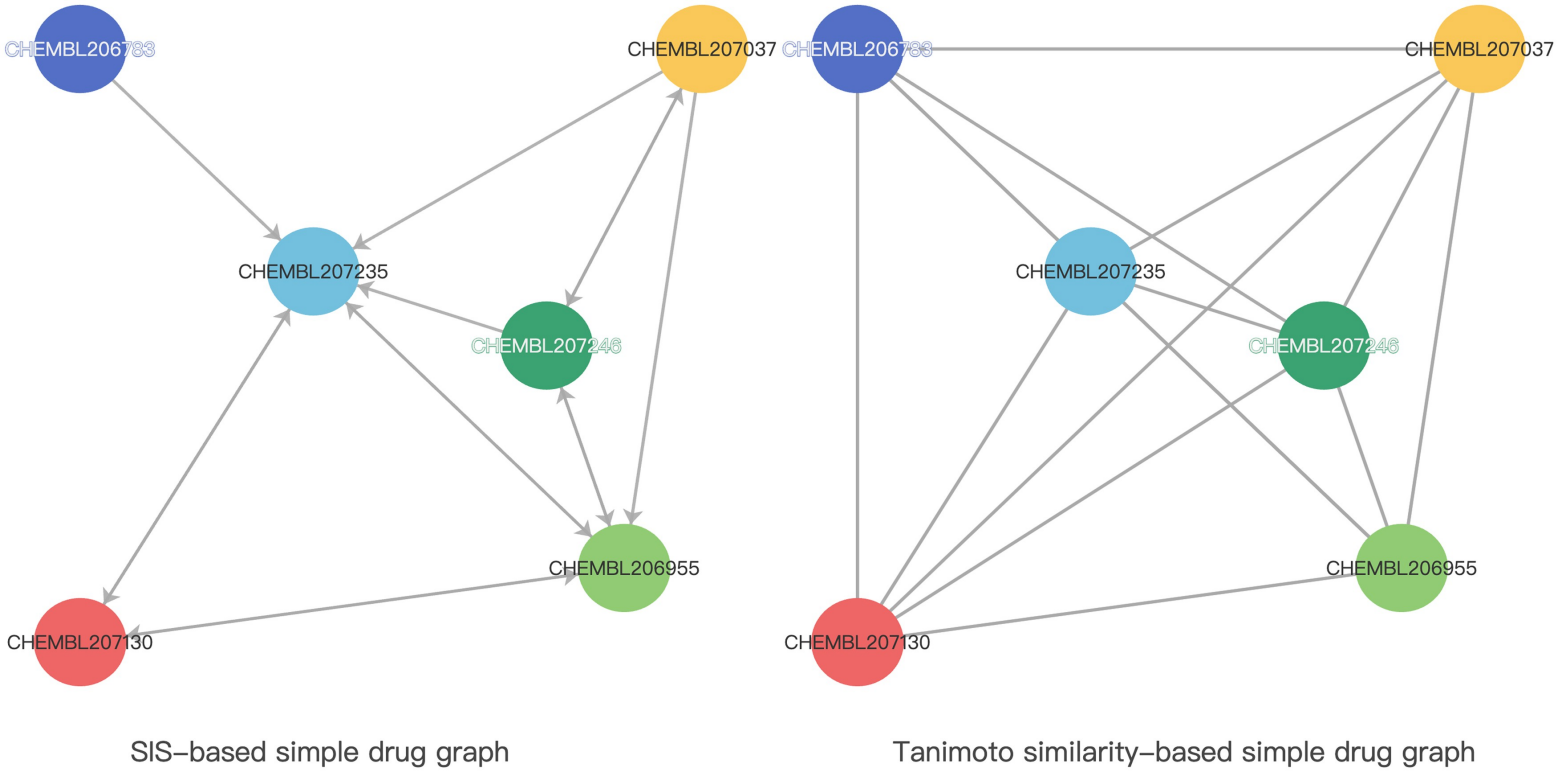
Comparison of composite relationship graphs generated using SIS and Tanimoto similarity. The graph based on Tanimoto similarity is represented as an undirected graph, with undirected edges in the graph representing two edges in both directions. Edges are generated when the similarity between two drugs is greater than a certain threshold. SIS-based graphs are represented as directed graphs, where the similarity between drugs is asymmetric. After setting a threshold, different similarities can result in an edge not existing, and the arrows indicate the direction of the edges that exist in the graph.

Although previous works have achieved milestones, some issues still need reinvestigating. Methods based on the ‘‘guilt-by-association’’ assumption (Cheng *et al.* 2012, Lu *et al.* 2017) usually use traditional similarity, resulting in graphical models with undirected associations between similar molecules. Figure 1 shows two simple drug graphs constructed using Tanimoto similarity and SIS, respectively. The SIS-based graph discards edges with less weight so that the edges of the graph have the direction. The graph based on traditional similarity makes symmetrical identical weight associations for two drug nodes. This simple association ignores the inclusion and compatibility of molecular structures. That is, the two drug nodes in the graph have the same similarity score and receive equally weighted information from the other. The intersecting parts of the substructures of the two drug nodes convey valid information. The remaining substructures beyond the intersection mostly contain only information that can represent the respective drug nodes. These substructures of one drug but not the other drug node contain the information that should be regarded as noise in the graph model. However, on undirected graphs constructed based on traditional similarity, they are also passed to each other with the same weight. A graph model constructed in this way would then have a significant amount of noise passing along the edges, which may affect prediction performance.

**Figure 2.**
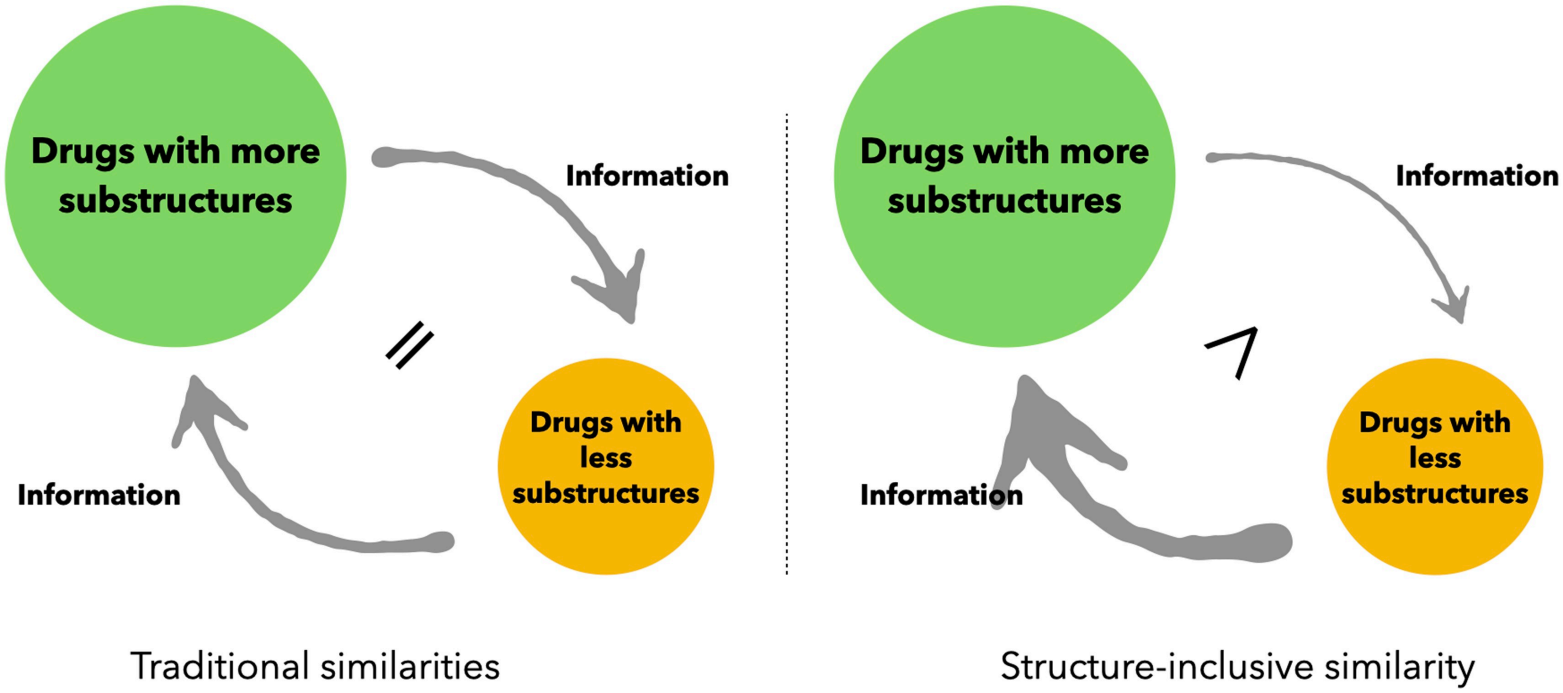
Traditional similarity versus SIS in message passing. Traditional similarity gives the same weight to both sides of a drug. In contrast, SIS assigns different weights to both sides of a drug based on the inclusion relationship of the substructure of both sides.

To explore the difficulty of intermolecular binding in more detail, we focus on predicting the more informative DTA. The biological activity of a molecule or any of its other properties can be explained by its substructures (Nicolotti *et al.* 2011). Certain privileged structures can even determine whether or not they will interact with certain targets (DeSimone *et al.* 2004). Considering the inclusion and compatibility relationship between molecular structures, we propose an SIS-based directed GNN model. Unlike other works, the similarity scores used to construct the graphical model are different in both directions. The scores for two drugs are calculated from the weight of the intersection of their substructures in all their respective substructures. Drugs with more substructures will have a smaller proportion of intersections in all substructures and thus receive a lower score. The more substructures beyond the intersection of a drug, the more likely it is to contain information about biological activity that another drug does not have. This information becomes noise when such a drug is linked to other drugs through edges in the graph model. The transmission of this noise is suppressed by assigning smaller weights to such drugs. Drugs with a smaller proportion of intersections also have relatively fewer substructures that the other drug does not have. This also leads to relatively more relevant bioactive information, which should be conveyed in the graph model. Higher weights will allow their useful information to be better conveyed in the graph model (Fig. 2 for details). The proportion of substructure intersections determines whether more useful information can be provided to each other. The directed nearest neighbor graph is built according to this principle to effectively improve the message-passing mechanism. Given that target classes other than the protein kinase family do not provide such rich DTI data, our work will focus on the protein kinase family. Our work achieves better results on mainstream datasets and the simplicity of the calculation can be extended to other methods using drug similarity.

## 2 Materials

We worked on the Davis (Davis *et al.* 2011) and KIBA (Tang *et al.* 2014) datasets. Both datasets contain kinase protein families and their inhibitors. He *et al.* (2017) screened the original KIBA dataset by removing all drugs and targets with low-level binding affinity and retaining 24% of them. The original Davis dataset had default values set for data with low or no known affinity. To avoid the influence of these default values on the model prediction results, MdeePred (Rifaioglu *et al.* 2021) removes these default values and proposes a filtered Davis dataset. The filtered Davis dataset retains 9125 known drug–target binding affinity data. Details of the three datasets are presented in Table 1.

**Table 1.** Summary of the datasets.

Dataset	Proteins	Compounds	Interactions
Davis	442	68	30 056
Filtered Davis	379	68	9125
KIBA	229	2111	118 254

SMILES is a molecular representation system designed for bioinformatics and is available for multiple computer services (Weininger 1988). Both datasets provide SMILES information for drugs, primary sequence information for proteins, and their respective similarity matrices.

The drug similarities are calculated by the similarity service provided by PubChem (Kim *et al.* 2021), and the target similarities are measured by the normalized Smith–Waterman method. In addition, we collated gene ontology (GO) information for targets in the form of bit vectors from the UniProt database (The UniProt Consortium 2023).

In this setup, we assume that there are unknown values in the provided drug–target binding affinity, and our main work is to make inferences about these unknown values. We have used the same distribution of training-test set as other works that have used the Davis and KIBA datasets to facilitate a more intuitive comparison.

## 3 Method

**Figure 3.**
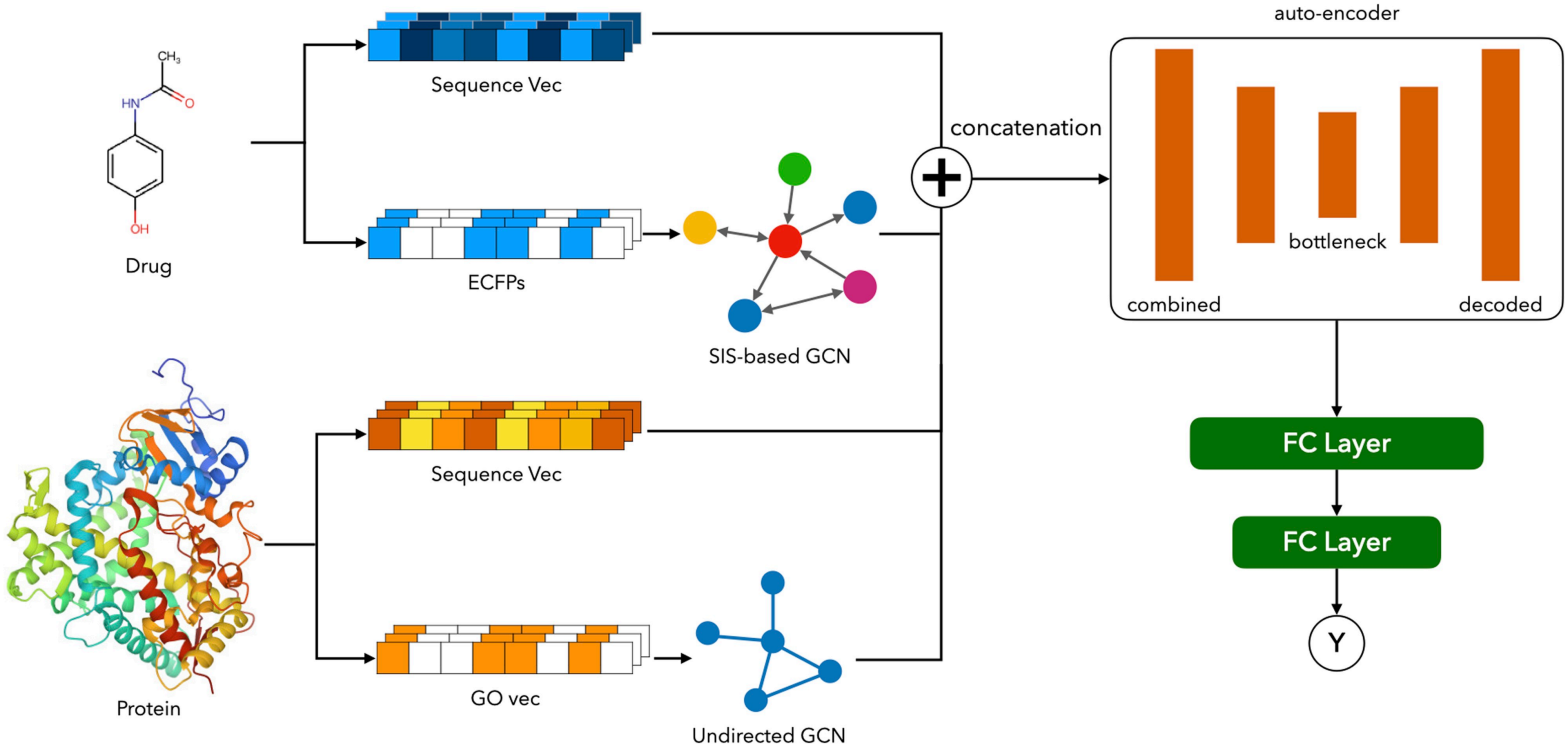
Our model learns nearest neighbor features from multiplexes for binding affinity prediction. Information on ECFPs for drugs was used to obtain structure-inclusive similarity and to construct directed graphs using it. The SW similarity was used to construct an undirected graph for the protein. The ECFPs of the drugs and GOs of the proteins were combined with their sequence information and input to AutoEncoder to be used to predict the final DTA.

We propose a deep-learning model incorporating GNN for DTA prediction. The input of the model is a drug–target pair, the output is its binding affinity score. In terms of feature usage, multiple features are considered simultaneously. A directed nearest neighbor graph is constructed based on structure-inclusive similarity, the weight in message passing is determined by calculating the proportion of the intersection of the two samples to the respective overall information separately. The graph neural network learns the drug and protein features and then combines them with their respective sequence features. The combined features are dimensionally reduced by AutoEncoder and handed over to the fully connected layer for processing. The loss function of the model consists of two components, MSE loss functions for label values *Y* and predicted values $\hat{Y}$, and MSE loss functions for AutoEncoder used for dimensionality reduction.

(1) $$\text{loss}=\frac{1}{q}\sum_{k=1}^{q}{\left(Y_{k}-{\hat{Y}}_{k}\right)}^{2}+\frac{1}{q}\sum_{k=1}^{q}{\left(H_{k}^{I}-H_{k}^{D}\right)}^{2}$$

where *q* is the number of drug–target pairs involved in the calculation, $H_{k}^{I}$ is the *k*th original sample feature, $H_{k}^{D}$ is the *k*th decoded sample feature. The pipeline of our model is shown in Fig. 3.

### 3.1 Drug representation

Extended connectivity fingerprints (ECFPs) are a molecular representation, which is generated by a ring-neighbor mapping algorithm. It has a simple structure and can form a fixed-length code regardless of the size of the molecule. SMILES contains the vast majority of information about the drug, but its string approach is not suitable for direct use in related work. Previous work has considered using ECFPs or Mol2Vec (Jaeger *et al.* 2018) for processing. ECFPs focus on indicating the presence or the absence of a certain substructure. We use RDKit to convert the SMILES of a drug into ECFPs of radius 4 and length 1024 and thus obtain structural information of the drug. Let $R=\{r_{1},r_{2},\ldots ,r_{m}\}$ represents *m* drugs in the dataset, whereby the ECFPs feature matrix $S_{fp}\in R^{m\times 1024}$ of the drug is obtained. Mol2Vec treats SMILES as sentences and substructures as words from the NLP perspective and applies the Word2vec algorithm to obtain composite vectors. The Mol2Vec method in the DeepChem (Ramsundar *et al.* 2019) package was used to generate the drug features according to its SMILES. The feature matrix of drugs was denoted by $S_{mv}\in R_{m\times 300}$. $S_{mv}$ is considered as the sequence information of drugs and is combined with the structural information to achieve the effect of complementing drug features.

### 3.2 Protein representation

Some work has attempted to use CNNs to process protein one-dimensional sequences (Öztürk *et al.* 2018, Nguyen *et al.* 2021), CNNs need to control the input to a fixed length. However, all proteins have different lengths, and sequences that are too long will be intercepted, resulting in partial loss of information. The UniProt database (The UniProt Consortium 2023) has more than 250 000 000 protein sequences, but the PDB (Berman *et al.* 2000) database contains only 215 401 protein tertiary structures, representing <1% of the protein sequences. These characteristics make protein tertiary structures limited to a certain extent. Therefore, we took the same treatment as the drug and performed feature extraction for it from multiple perspectives of features at the same time.

Gene ontology (GO) is an important bioinformatics initiative that aims to represent gene and gene product properties (Gene Ontology Consortium 2008) uniformly for all species. Each of the gene ontology annotations provides a biological account of the gene, including the molecular functions, cellular locations, and biological processes. The UniProt database (The UniProt Consortium 2023) provides gene ontology annotations for individual proteins. We set the gene ontology information obtained from the UniProt database into columns, one protein per row and label it, with the column labeled 1 if the protein contains a particular gene annotation and 0 otherwise. All the gene ontology information are mapped into gene ontology vectors which can be delivered to downstream tasks very simply. Let $P=\{p_{1},p_{2},\ldots ,p_{n}\}$ represents the *n* proteins in the dataset, $S_{go}\in R^{n\times l}$, where *l* is the number of GO annotation categories involved in all proteins in this dataset. the matrix is shaped like the ECFPs feature matrix, where a protein contains a certain gene annotation then the column is 1, otherwise, it is 0. For protein sequences, in order to avoid the problem of information loss caused by CNN directly intercepting one-dimensional sequences, information is obtained on the basis of BERT. We convert the sequences into vectors of length 1024 by ProtTrans (Elnaggar *et al.* 2022) to obtain the matrix $S_{pt}\in R^{n\times 1024}$.

### 3.3 Structure-inclusive similarity

In this study, we propose a structure-inclusive similarity (SIS)-based computation method to construct bivariate similarities with different sizes for drug pairs. The SIS is calculated as follows:

(2) $${\text{SIS}}_{i,j}=\frac{\left|r_{i}\cap r_{j}\right|}{\left|r_{j}\right|},$$

where ${\text{SIS}}_{i,j}$ is the inclusive similarity of drug $r_{i}$ to $r_{j}$, that is, the amount of information that drug $r_{i}$ can collect from $r_{j}$. Here, we use the structural features (for drugs as ECFPs) to calculate the inclusive similarity.

From a bioinformatics point of view, substructures can represent how much information is available. Suppose the substructures of drug $r_{i}$ and drug $r_{j}$ have intersections, and the substructures outside the intersection of each drug are called their respective complements. If the intersection is in a larger proportion of drug $r_{j}$, then drug $r_{i}$ contains more substructures of drug $r_{j}$. The information contained in this molecular structure can be used by drug $r_{i}$. Drug $r_{j}$ has a complement of substructures that drug $r_{i}$ does not possess, and the information contained in these substructures should be regarded as noise. However, these substructures are smaller than the substructure complement of drug $r_{i}$, so the amount of noise is relatively small. Therefore, drug $r_{i}$ can obtain more information from drug $r_{j}$ and can obtain a higher similarity score.

**Figure 4.**
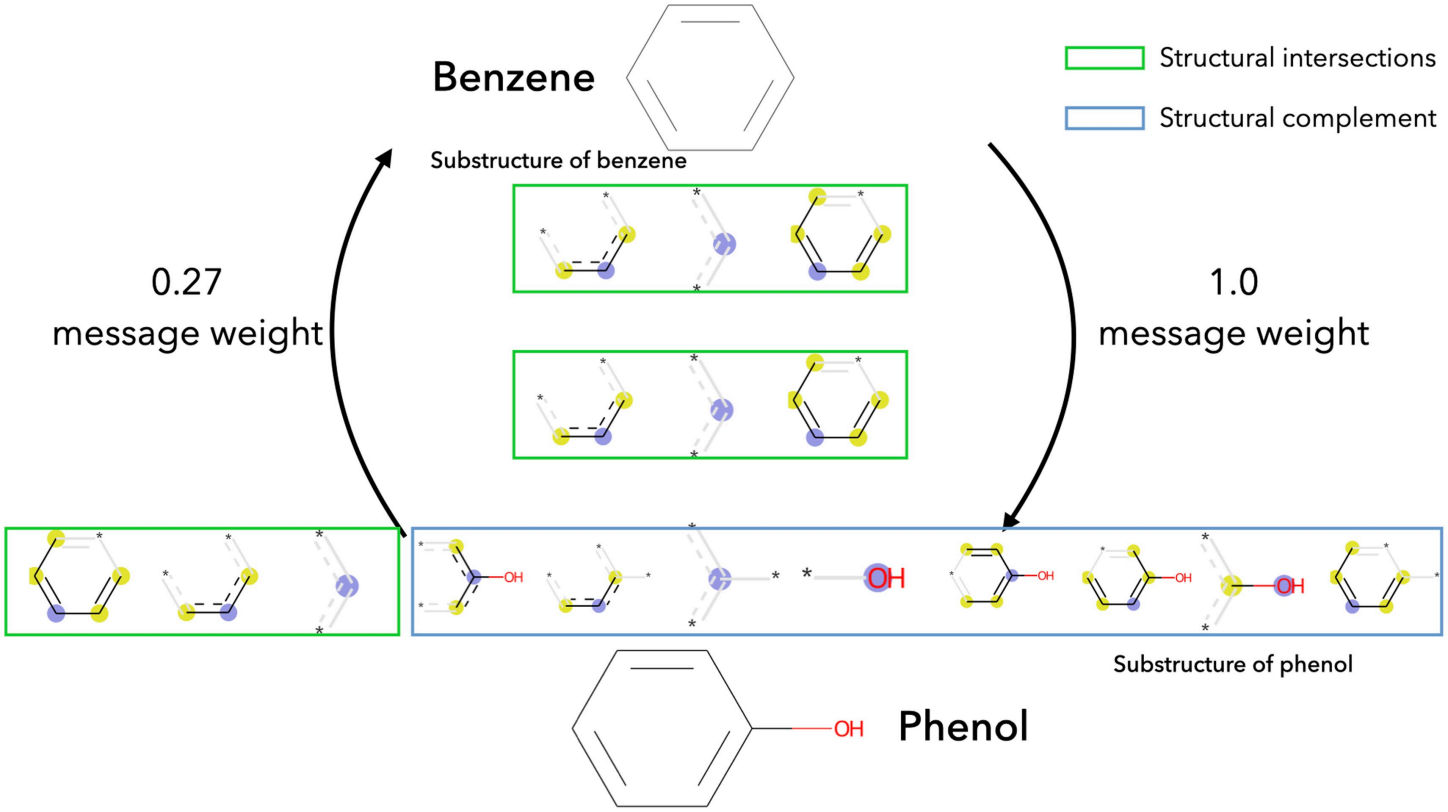
Generating similarity between benzene and phenol, based on SIS similarity. The substructure phenol of benzene is owned in its entirety and can be given a message weight of 1.0. Structures with hydroxyl groups in phenol, do not carry information useful for benzene.

Relatively, if structural intersection only has a small proportion of the drug $r_{i}$, it indicates that the valid information provided by the drug $r_{i}$ is only a very small part of itself. The noise from the complement of its substructure is relatively dominant and cannot be given high weight in the message passing. For example, benzene and phenol, phenol has most of the properties of benzene and can undergo halogenation, oxidation, and other reactions. Phenol itself, on the other hand, has properties such as being acidic and more susceptible to substitution reactions than benzene due to its hydroxyl group. As shown in Fig. 4, benzene contains three substructures and phenol contains 11 substructures. Of these, phenol possesses all the substructures of benzene and the remaining substructures of phenol are all associated with hydroxyl groups. As described above, the hydroxyl group possessed by phenol is the main reason for the difference, and the biological information it carries does not apply to benzene. In terms of drug repurposing, assuming the presence of undiscovered biological properties for phenol, information for benzene could be obtained to make inferences. Conversely, there are many substructures of phenol that benzene does not possess. The biological information brought by the hydroxyl group would be perceived as noise by benzene, making it difficult to infer effective biological properties from phenol.

Corresponding to message passing, phenol should get all of the benzene’s information while benzene should get a small amount of phenol’s information. However, when calculating using traditional similarity, both would obtain a similarity score of 0.27 (based on Jaccard similarity). In this case, phenol is unable to obtain information such as chemical reactions related to benzene, and the prediction model will therefore have reduced performance. After calculations using SIS, the information weight from benzene to phenol is 1.0 and from phenol to benzene is 0.27. When constructing the graph, edges with low weights can be discarded to prevent noise from other substructures of phenol from being passed to benzene.

### 3.4 GCN-based drug and target embedding vector learning

In drug–target relationship prediction problems, many models involving the use of similarity are mostly based on the ‘‘guilt-by-association’’ assumption (Thafar *et al.* 2019). Obtaining information from similar nearest neighbors to make predictions is a very common approach. In our work, the GCN model (Kipf and Welling 2016) is chosen to obtain information about the nearest neighbors of drugs and proteins.

In general form, the set of drugs and the set of proteins in the dataset are represented as a graph of the shape G=(V,E), where *V* is the set of nodes, representing either drugs or proteins, each represented by a *d*-dimensional vector, *E* is the set of edges, representing two nodes having some degree of similarity, represented by the matrix ***A***. Each GCN layer accepts the set of input samples $X\in R^{N*d}$ (N=|V|) to obtain the convolutional output $X^{\prime }\in R^{N*k}$ for that layer. The specific form is

(3) $$X^{\prime }={\hat{D}}^{-1/2}\hat{A}{\hat{D}}^{-1/2}X\Theta .$$

Unlike the matrix *A* which only uses {0,1} to represent the presence of edges, we use the adjacency matrix $\hat{A}$ which contains the edge weights. ${\hat{D}}_{ii}=\sum_{j=0}{\hat{A}}_{ij}$ is the pairwise angle matrix, and $\Theta \in R^{d*k}$ is the learnable parameter matrix, here *d* represents the dimension of the features provided by the previous layer of the network and *k* represents the dimension of the features output by this layer of the network.

In our work, both drugs and proteins use GCN models to learn information about their nearest neighbors. Using SIS similarity scores as the weights of edges for drugs, and Smith–Waterman similarity scores as the weights of edges for proteins. In traditional similarity construction graphs, the similarity scores of two nodes are symmetric. If there exist two nodes $\{v_{i},v_{j}\}\in V$ and the similarity is greater than a threshold δ, it will set both $A_{i,j}$ and $A_{j,i}$ to 1 in the adjacency matrix *A*. All these edges are symmetric and have the same weight, and are reflected as undirected graphs. When constructing the graph using the SIS, the weights differ between the two nodes. The values in the adjacency matrix $\hat{A}$ formed at this point are set to the weights of the edges, and these weights are not all the same. In this case, we consider discarding some of the edges with weights less than the threshold δ. For example, $\omega_{i,j}$ represents the similarity (weight) of nodes $v_{i}$ to $v_{j}$. If $\omega_{i,j}<\delta$, then discard the edge $v_{i}$ to $v_{j}$. The message from node $v_{i}$ cannot be passed to node $v_{j}$, controlling the direction of the message passing. The edges left by this calculation method are those that can provide effective information, and the discarded edges also suppress the transmission of noise. The constructed graph is as shown in Fig. 1, after removing some edges, the overall information flow shows the direction.

**Figure 5.**
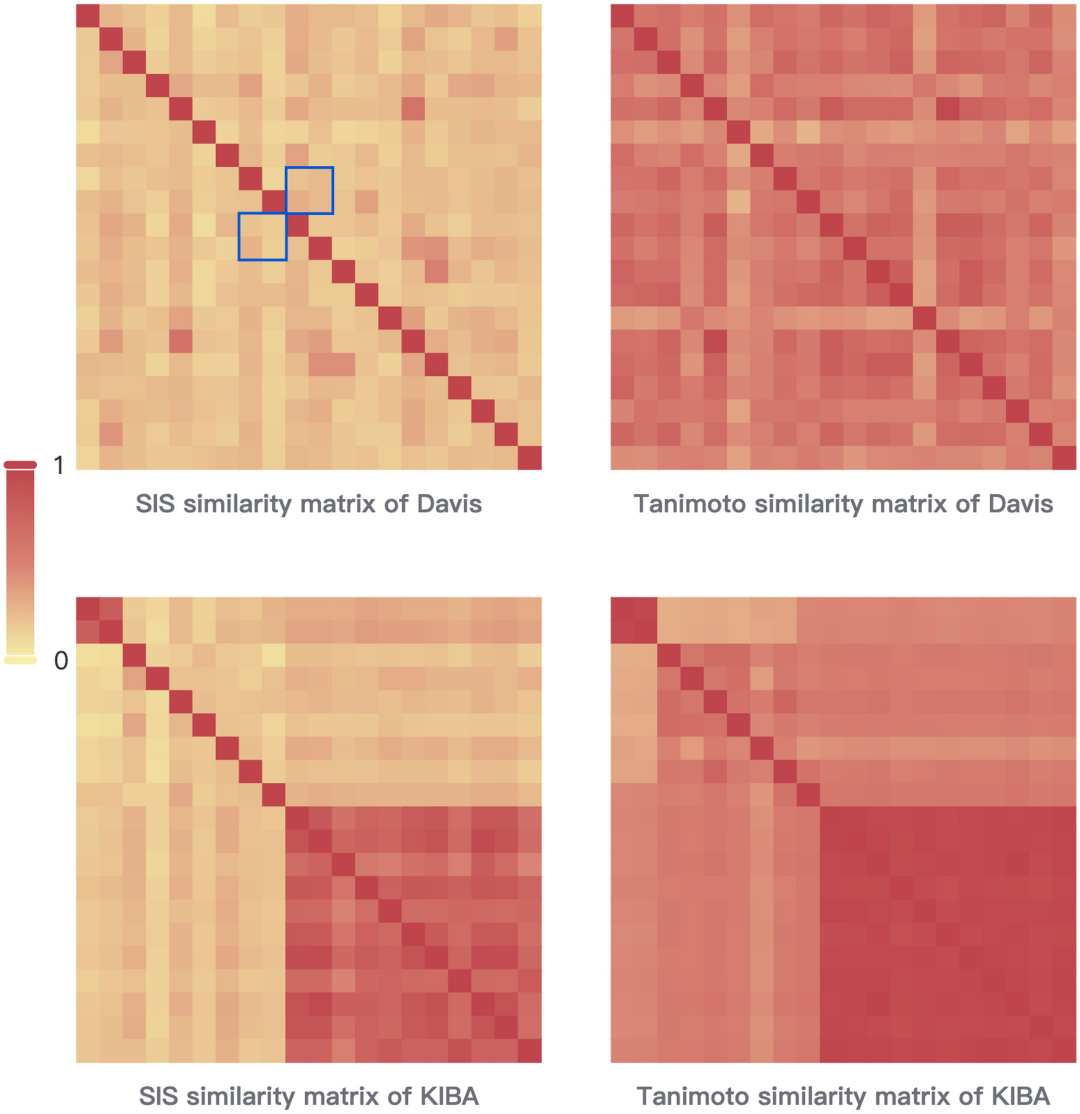
The similarity heat map of the top 20 compounds under the Davis and KIBA datasets. The similarity matrices constructed by conventional similarity are embodied as symmetric matrices, whereas the SIS matrices are embodied as asymmetric matrices. As shown in the box with borders, the similarities within the box are not symmetrical.

It is worth noting that when constructing a graph based on traditional similarity, undirected edges are symmetrical between two nodes. In contrast, when constructing the graph based on SIS, the similarity between the two nodes is no longer symmetrical. In this case, setting the threshold will result in two nodes not necessarily having symmetric edges, and these one-way edges make the graph a directed graph. This is also reflected in the heat map of similarity (Fig. 5). The heat map of the traditional similarity matrix has the overall matrix in a symmetric form. There is no difference in the validity of the amount of information between the two sides, nor is any distinction made between potential noise. With the use of SIS, the overall similarity matrix can be seen in the heat map as no longer symmetrical. The asymmetric similarity can make a distinction between the proportional size of the noise and improve the overall message passing in the graph.

The improvement is even more pronounced with the extension of single-layer GNNs to multi-layer GNNs. Multilayer GNNs based on undirected graphs also consider the nearest neighbors of neighboring nodes when aggregating information. This allows noise to also be passed across layers in the graph. In the SIS-based directed graph, the removed one-way edges reduce the transmission of noise to the neighbors. The extension to a multi-layer GNN eliminates the transmission of this noise to the outer layers. After aggregating the sparse features consisting of these bit vectors, AutoEncoder is used to reduce the dimensionality of these features and speed up the subsequent operations of the model.

### 3.5 Construction of the relationship graph

The strategy for constructing the graph is also important to make relationship graph-based messaging more efficient. Many traditional similarity-based tasks use a simple approach: If the similarity score of two drugs is >0.5, it means that there is some relationship between the two drugs; similarly, if it is <0.5, no relationship is established between the two drugs. This approach is not directly applicable under the SIS strategy. As in the example of benzene and phenol noted above, the traditional similarity scores and SIS can differ, and using the same similarity threshold may result in some relationships being ignored or some relationships being incorrectly added. We thus chose different similarity thresholds depending on the task, thus enabling the model to be better adapted to different tasks. In this work, we use multiple similarity thresholds to train the model separately, and finally choose the one that works best.

**Figure 6.**
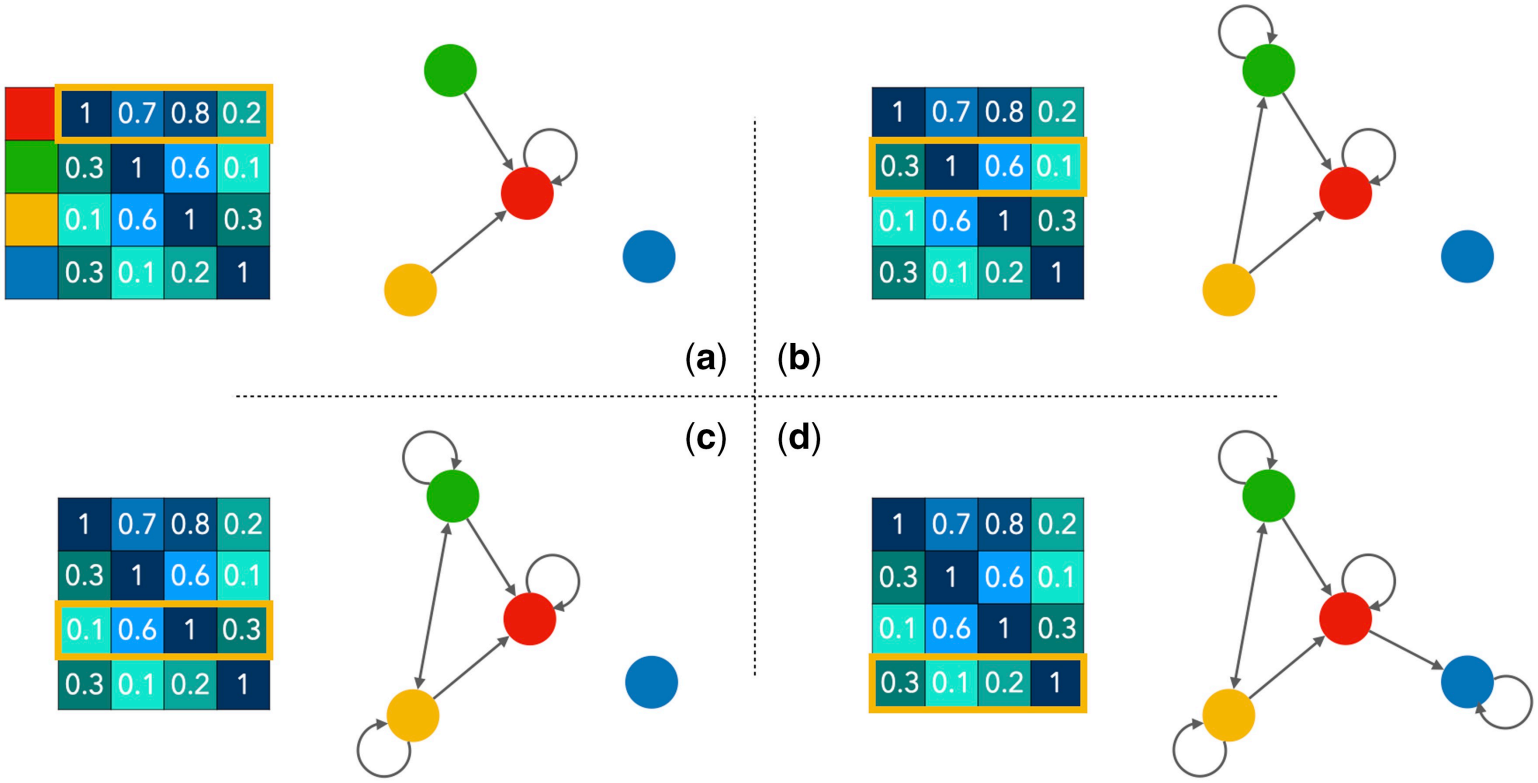
Schematic representation of the steps to construct a relationship graph of four nodes based on the similarity matrix of the SIS strategy. a, b, and c show the process of constructing edges using the similarity scores of each and other nodes, respectively. The boxes with borders represent the similarity scores used in the current step, where a relationship between two nodes is considered to exist if the threshold is >0.6. In step d, there are no eligible similar nodes, and the first *k* most similar neighbors are taken to build the edge, with *k* being 1 in this illustration.

During the construction of relationship graphs based on thresholds, it often happens that certain nodes have a degree of 0, i.e. no edges are connected to them. Corresponding to drug relationship graphs, these nodes represent orphan drugs that have no similar drugs. These drugs do not have any other drugs connected to them and no information about other drugs can be learned in the graph network. This situation can result in the features of these nodes not being updated during subsequent message passing, thus affecting the prediction of the model. To improve this situation, we added other operations after the drug association based on thresholds. If the number of neighboring nodes for the drug is less than *k*, it is associated with the most similar other drugs to bring the number of neighboring nodes to *k*. At the same time, these additional neighboring nodes still maintain their lower similarity weights and the incoming noise is controlled. In this work, we set the value of *k* to 5 to ensure that each drug is available from other similar drugs. Figure 6 shows an example of a relational graph with four nodes, a threshold of 0.6 and *k* of 1.

Tables 2 and 3 show the effect of different threshold settings on the prediction results in the two datasets. When the threshold is too small, the number of neighboring nodes for each drug node is too high, resulting in too much complexity in the model’s message passing. Each drug node also acquires more noise, affecting the prediction performance of the model overall. When the threshold is too large, the number of neighboring nodes per drug node is too small, a large number of orphan nodes appear in the graph, and the overall degradation to a graph network of *k*-nearest neighbors, and the prediction performance of the model suffer. In the KIBA dataset, the best prediction performance of the model was achieved with a threshold value of 0.7. In the Davis dataset, a threshold of 0.6 would give the best results for the model.

**Table 2.** Comparison of prediction performance with different similarity thresholds in drug relationship graph under Davis dataset.^a^

Threshold	MSE	CI	$r_{m}^{2}i\mathrm{ndex}$
0.4	0.172942	0.906392	0.767983
0.5	0.171238	0.905109	0.768230
0.6	**0.169386**	**0.907412**	**0.771037**
0.7	0.172072	0.902630	0.761947

a The best performing set of experimental results are highlighted in bold.

**Table 3.** Comparison of prediction performance with different similarity thresholds in drug relationship graph under KIBA dataset.^a^

Threshold	MSE	CI	$r_{m}^{2}i\mathrm{ndex}$
0.5	0.127521	0.897451	0.794312
0.6	0.125166	0.897052	0.806611
0.7	**0.122451**	**0.902132**	**0.809529**
0.8	0.124528	0.898935	0.801295

a The best performing set of experimental results are highlighted in bold.

## 4 Results

### 4.1 Experimental comparison

As baselines for our results, existing models that also work on the Davis and KIBA datasets were selected for comparison. we used MSE, CI (Gönen and Heller 2005) and $r_{m}^{2}i\mathrm{ndex}$ (Pratim Roy *et al.* 2009) as performance metrics to evaluate the model. To facilitate and visualize the comparison, we used the same training-test set distribution, performance metrics, and the same approach to affinity values as these excellent works, as shown in detail in Table 4. On the Davis dataset, the MSE is reduced by 0.026 over the best baseline, and the CI metric and the $r_{m}^{2}i\mathrm{ndex}$ metric are improved by 0.001 and 0.023, respectively.

**Table 4.** The results of the comparison of prediction performance on the Davis and KIBA datasets, with the best performance indicated in bold.

Dataset	Davis			KIBA		
Model	MSE	CI	$r_{m}^{2}i\mathrm{ndex}$	MSE	CI	$r_{m}^{2}i\mathrm{ndex}$
KronRLS (Pahikkala *et al.* 2015)	0.379	0.871	0.407	0.411	0.782	0.342
SimBoost (He *et al.* 2017)	0.282	0.872	0.655	0.222	0.836	0.629
DeepDTA (Öztürk *et al.* 2018)	0.261	0.878	0.630	0.194	0.863	0.673
GraphDTA (CNN+GCN) (Nguyen *et al.* 2021)	0.254	0.880	–	0.139	0.889	–
GraphDTA (CNN+GAT) (Nguyen *et al.* 2021)	0.232	0.892	–	0.179	0.866	–
GraphDTA (CNN+GIN) (Nguyen *et al.* 2021)	0.229	0.893	–	0.147	0.882	–
GraphDTA (CNN+GAT–GCN) (Nguyen *et al.* 2021)	0.245	0.881	–	0.139	0.891	–
MATT_DTI (Zeng *et al.* 2021)	0.229	0.890	0.682	0.150	0.889	0.756
MgraphDTA (Yang *et al.* 2022)	0.207	0.900	0.710	0.128	0.902	0.801
BiComp-DTA (Kalemati *et al.* 2023)	0.237	0.904	0.696	0.168	0.891	0.757
FMDTA (Zhang *et al.* 2023)	0.195	0.906	0.748	0.133	0.899	0.801
Our work	**0.169**	**0.907**	**0.771**	**0.122**	**0.902**	**0.809**

On the dataset KIBA, our work remains the best performance. Our study shows a decrease of 0.015 from the best baseline in MSE, and an improvement of 0.008 in the $r_{m}^{2}i\mathrm{ndex}$ metric, respectively.

To ensure fairness in the comparison, we have selected models that have been fine-tuned in the filtered Davis dataset for comparison. As shown in Table 5, our work still achieves the best results without additional fine-tuning.

**Table 5.** Comparative results for the predictive performance of the filtered Davis dataset, with the best performance highlighted in bold.

Model	RMSE	CI	Spearman
MDeePred (Rifaioglu *et al.* 2021)	0.742	0.733	0.618
CGKronRLS (Airola and Pahikkala 2017)	0.769	0.740	0.643
DeepDTA (Öztürk *et al.* 2018)	0.931	0.653	0.430
MGraphDTA (Yang *et al.* 2022)	0.695	0.740	0.654
Our work	**0.656**	**0.773**	**0.727**

### 4.2 Ablation experiment

**Figure 7.**
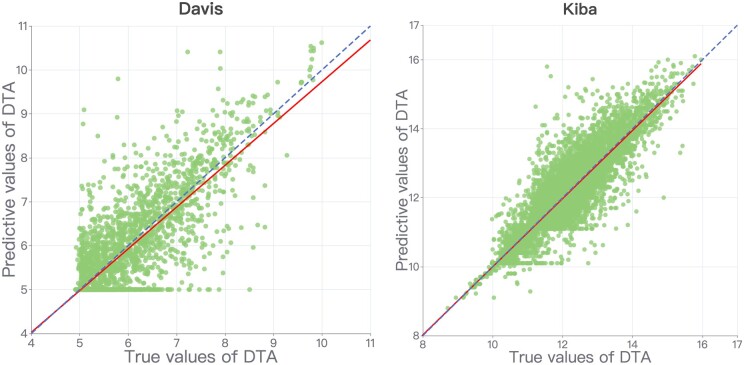
Predicted and true values for the Davis dataset and the KIBA dataset. The coordinate axes represent the DTA scores, where the dashed line represents the true value equal to the predicted value. The solid line represents the fitted regression line between the true and predicted values.

Figure 7 shows the DTA of the true and predicted values on the two datasets, where the dashed line represents the perfect prediction case where the predicted value is equal to the true value. It can be observed that the data are more scattered in the Davis dataset, which has fewer data. While in the KIBA dataset, which has more data, the data are mostly concentrated around the perfect prediction line.

We chose the KIBA dataset with higher data density for the ablation experiments. As shown in Table 6, combining the sequence features of drugs can improve the performance of the model, and the SIS similarity outperforms the traditional similarity in several evaluation metrics. These results show that SIS can improve the defective information transfer between samples from different perspectives, avoid ineffective bidirectional edges in the nearest-neighbour graph, and improve the information-acquisition ability of the model.

**Table 6.** Ablation experiments for drug feature under the KIBA dataset.^a^

Sequence features	Structural features	MSE	CI	$r_{m}^{2}i\mathrm{ndex}$
Word2vec	Not set	0.167	0.828	0.669
Not set	ECFPS	0.146	0.839	0.738
Not set	ECFPS (SIS-based GCN)	0.131	0.892	0.781
Word2vec	ECFPS	0.149	0.845	0.736
Word2vec (SIS-based GCN)	ECFPS	0.147	0.851	0.762
Word2vec (SIS-based GCN)	ECFPS (SIS-based GCN)	0.140	0.887	0.775
Word2vec	ECFPS (Tanimoto-based GCN)	0.135	0.890	0.779
Word2vec	ECFPS (Euclidean-based GCN)	0.126	0.899	0.799
Word2vec	ECFPS (Jaccard-based GCN)	0.125	0.897	0.800
Word2vec	ECFPS (SIS-based GCN)	**0.122**	**0.900**	**0.809**

a The best performing set of experimental results are highlighted in bold.

Also, we compare both datasets by using the Simboost model. Only the similarity strategy used for the graphs in the model was replaced and the rest of the model was left unchanged. SimBoost uses traditional similarity in constructing predictive models to construct graphs. After replacing the traditional similarity with SIS, tests were conducted on both datasets. The division of the training and test sets was performed randomly, and the mean was recorded for multiple tests. As can be seen from the results in Table 7, SIS is effective in improving the prediction results.

**Table 8.** Top 10 candidate drug−target pairs.

No	Drug	Protein	PubChem bioassay record	Activity type	Activity value
1	CHEMBL249097	P07947	389065	Kd	0.001 μM
2	CHEMBL1996510	Q04759	690003	IC50	0.0005 μM
3	CHEMBL1969151	O00311	673702	Ki	0.0003162 μM
4	CHEMBL523586	Q9Y243	417621	IC50	0.002 μM
5	CHEMBL1241676	P08631	507080	IC50	0.00018 μM
6	CHEMBL478629	P36888	527867	IC50	0.001 μM
7	CHEMBL2004290	P17948	1800448	IC50	0.00429 μM
8	CHEMBL178737	P49841	241844	IC50	0.0013 μM
9	CHEMBL1980995	Q04912	None	–	–
10	CHEMBL2003638	O00311	493040	Ki	0.000631 μM

### 4.3 Case studies

We use a model based on the SIS to make predictions of binding affinity scores for other unlabeled compound–target pairs and attempt to validate the reliability of these results. We performed prediction validation on the newer KIBA dataset, Table 8 lists the top 10 compound-target candidates with the highest scores in the prediction results.

**Table 7.** Modify different similarities in the Simboost model to construct graphs for comparison.^a^

Similarity strategy		Davis MSE	KIBA MSE
SimBoost	Tanimoto	0.289(0.005)	0.296(0.003)
SimBoost	Jaccard	0.275(0.002)	0.283(0.001)
SimBoost	SIS	**0.274(0.003)**	**0.271(0.003)**

a The best performing set of experimental results are highlighted in bold.

PubChem (Kim *et al.* 2021) is an open chemistry database from the National Institutes of Health (NIH) that provides a lot of chemistry-related information. As shown in Table 8, 9 of the top 10 candidates with high prediction scores can be found in this database with relevant validation. This is also a side note that the unassayed compound CHEMBL1980995 may have a potential drug–target relationship with the target Q04912.

## 5 Conclusion

In this study, we propose a directed similarity calculation strategy to improve the message-passing mechanism in the GCN-based DTA prediction model. SIS can measure the similarity of two drugs by considering the inclusion relationship of their substructures. We also developed a GCN-based DTA prediction model according to the drug-similar graph and target-similar graph constructed by SIS. Experimental results show that SIS is superior to a variety of other traditional undirected similarities. We tested our SIS-based DTA prediction method on both KIBA and Davis datasets and compared it with several baselines. The results showed that our method is more accurate for DTA prediction and outperforms all the baselines in various evaluation metrics. Case studies and the docking results further confirm that our method is strongly for predicting the binding affinities between drugs and targets.

Previous studies have shown that “guilt-by-association” is applicable in multiple tasks such as miRNA−disease relationship prediction (Chen *et al.* 2019, 2021) and drug−disease association prediction (Xuan *et al.* 2022). As a reliable similarity evaluation metric, SIS is also suitable for these tasks.

## Data Availability

All data and codes used for this study are available online through: https://github.com/HuangStomach/SISDTA.
